# Singular entry point technique for forehead and temple filler augmentation: Anatomical and clinical perspectives

**DOI:** 10.1111/jocd.16411

**Published:** 2024-06-04

**Authors:** Hyo‐Sang Ahn, Hyoung Moon Kim, Wook Oh, Kyu‐Ho Yi, Jisoo Kim, Hee‐Jin Kim

**Affiliations:** ^1^ Division in Anatomy and Developmental Biology, Department of Oral Biology Human Identification Research Institute, BK21 FOUR Project, Yonsei University College of Dentistry Seoul Korea; ^2^ Maylin Clinic Goyang Gyeonggi‐do Korea; ^3^ Maylin Clinic Seoul Korea; ^4^ Maylin Clinic Apgujeong Seoul Korea; ^5^ Dr Youth clinic Seoul Korea; ^6^ Department of Electric and Electronical Engineering, College of Engineering Yonsei University Seoul Korea

**Keywords:** filler injection, forehead, minimally invasive procedures, Temple, ultrasonography

## Abstract

**Background:**

The depressed volume of the forehead and temple is resolved by filler injection. However, the current method has the potential to cause pain and side effects in patients, depending on the skill of the clinician. Therefore, this study proposes a new method for safer and simpler injection using only one injection entry point.

**Methods:**

Using the novel injection method, the filler was injected into the forehead and temple regions in three unembalmed cadavers and two healthy Korean volunteers. The cannula and filler locations were identified using dissection, ultrasonography, and three‐dimensional (3D) scanning.

**Results:**

Ultrasonographic images and dissection results showed that the filler injected into the cadavers was in the target layer. The cannula and filler were located on the layer as the supraperiosteal layer on the forehead and the supra deep temporal fascia layer in the temple. Finally, 3D scanning images showed that the filler was injected precisely and effectively into the forehead and temples of the volunteer who underwent the procedure.

**Conclusions:**

This method can reduce pain and minimize externally visible wounds caused by injections. The injected filler was naturally connected from the forehead to the temple and maintained for around 3 months. Additionally, it is possible to inject fillers into the forehead and temple at a constant and safe depth without requiring specific skills. It is expected that this method will become a universal method because it minimizes the burden on both patients and clinicians.

## 1 INTRODUCTION

The anatomy of the face has been studied by many researchers for a long time. For efficiency, researchers have classified the face into several regions, which naturally led to the classification of the face anatomical structures as regions. Anatomical structures like muscles, that have a relatively clear shape may be meaningful to be categorized. However, despite the ambiguous boundaries, facial tissues, such as fat, are sometimes defined and labeled as a single structure based on the region of the face.^1^, ^2^ Thus, our previous work questioned the existing definition of anatomical structures and was able to explain the partially.

One of our previous studies showed anatomically the relationship between the retro‐orbicularis oculi fat (ROOF) and the sub‐superficial musculoaponeurotic system (SMAS) fat, revealing them as an interconnected structures.^3^ ROOF is a representative loose alveolar connective tissue located in the forehead region, directly above the periosteum. ROOF was considered an independent fatty structure; however, according to our study, it is connected to surrounding structures, especially in the temple region. The loose connective tissue, which is connected to the ROOF in the temple region, exists between the SMAS and the deep temporal fascia (DTF). This connected structure was redefined as subSMAS fat, which was previously referred to as innominate fascia.

As like our former finding, understanding the individual anatomical structures of the face and relationship between them is clinically important. The forehead and temple are representative regions that show significant changes depending on age or body type. An undulated forehead and depressed temple may provide an aged and tired impression. Thus, filler augmentation procedures have been widely performed to improve this phenomenon in most cases.^4^ Previous studies have suggested that injection procedures can create a natural and smooth curvature of the forehead and temple.^5^

For forehead contouring, clinicians mainly inject fillers in three or four horizontal sites from the medial to the lateral side of the forehead or in various lateral directions using the fanning technique from one midline entry point on the central forehead.^6^, ^7^, ^8^, ^9^, ^10^ In the case of temple volumizing, efficient injection methods considering anatomical structures are being improved through continuous research, and currently the following two methods are most popular^10^, ^11^, ^12^, ^13^, ^14^: (1) injecting by points several times while carefully avoiding blood vessels, such as superficial temporal artery (STA),^15^ after determining the range of temple volumizing broadly based on the front of the hairline, or (2) injecting anteriorly to the medial aspect of the eyebrow. During filler injection, the location of the entry point and layer into which the filler is injected should be carefully determined. For effective superficial augmentation, the filler should be injected generally in the supraperiosteal layer of the forehead and in the layer between the DTF and the subSMAS fat of the temple. These methods require considerable attention and skill of clinicians. Furthermore, as this injection methods use multiple entry points, the pain and conspicuous injection marks could be a possible burden for the patients. This is the reason for the need of a new injection method that reduces pain in patients and make a low risk of side effects.

Recently, some clinicians have started using a new injection method, applying the results of anatomical study of ROOF and subSMAS fat. This new injection method allows filler to be injected into both the forehead and temple using only one injection entry point. This could solve the problem that the old method has many injection entry points and is difficult to accurately inject into the target layer, and could provide a very satisfactory procedure experience to both the patient and the clinicians. However, since this method is based on clinical experience, it needs to be verified for its safety and accuracy through anatomical consideration. Based on previous anatomical findings—connection of the forehead and temple areas by a single fat layer, ROOF and subSMAS fat—the aim of this study is to prove the accuracy and safety of the new injection method.

## 2 MATERIALS AND METHODS

Three hemifaces of three fresh frozen cadavers (two males and one female; mean age, 92.5 years) legally donated to Yonsei University Medical Center were used in this study. In all specimens, a filler (Lorient No. 4; Joonghun Pharmaceutical, Seoul, Korea) mixed with a dye (Davidson® Tissue Green Dye, #3408–1; Bradley Products, Indiana, United States) was injected into the forehead and temple regions.

Two healthy Korean volunteers (2 women; mean age 23 years) participated in the filler procedure. The volunteers had not received any noninvasive treatment or surgical procedures in the forehead and temple within the previous 6 months. Volunteers were injected with the same filler used in the cadaver study into the same regions.

This study was approved by the Institutional Review Board (IRB) of Yonsei University (IRB no. 2–2021‐0078), and all examinations were performed in accordance with the principles of the Declaration of Helsinki. Appropriate consent and approval were obtained from the families of the cadavers before the dissection was performed. The institutional ethics committee approved the study, and the volunteers provided written informed consent after the study had been fully explained to them.

### 2.1 Ultrasound‐guided injection using ultrasonographic scanning

The forehead and temple anatomical structures of cadavers and volunteers were scanned using an ultrasonographic (US) imaging system (Sonimage HS1; Konica Minolta, Tokyo, Japan). Through US scanning, the muscle structures, vessels, and layers of the forehead and temple to be injected with the filler were identified, and the changes in each structure were observed following filler injection. Based on the anatomical structure identification, US‐guided injection was performed for filler augmentation on both the forehead and temple in each cadaver. The entry point was approximately 1 cm above the frontotemporale, which is a craniometric landmark, along the superior temporal fusion line (STL) (Figure 1). The injection plane for the filler products using an 18‐ or 21‐G cannula was at the level of the supraperiosteal layer, between the periosteal layer and the ROOF, on the forehead and supra‐DTF layer, between the deep temporal layer and the subSMAS fat, on the temple. As injecting the filler, the cannula was inserted with scratching the surface of each layer after touching the periosteal layer and DTF layer of the forehead and temple, respectively. In this process, as these two layers are relatively hard and very elastic, the possibility of damage by the cannula is very low. During the injection, the US imaging system was used to detect the structures of the injection site and the position of the cannula. The US imaging system was set to a 18‐MHz B‐mode operation, and an L18‐4 transducer was used to acquire images.

**FIGURE 1:**
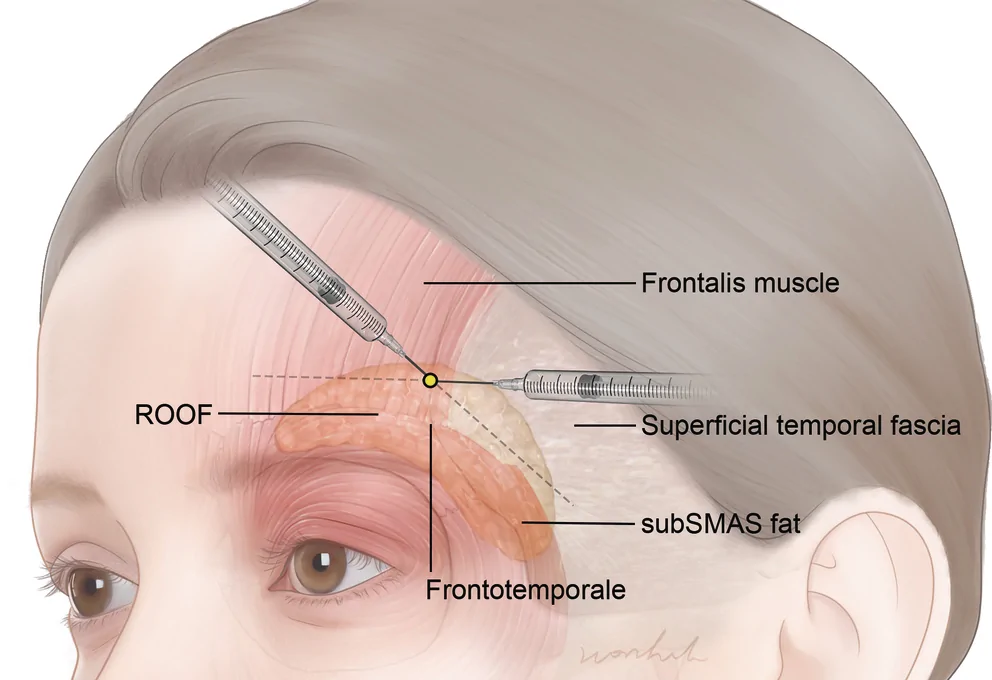
Filler injection techniques for the forehead and temple using cannula. Yellow point is the new entry point for filler injection.

### 2.2 Dissection protocol

After injection, the injected forehead and temple regions were dissected layer‐by‐layer to identify the layer containing the dye‐mixed filler. Dissections were performed to expose the frontalis muscle and the SMAS of the forehead and temple. An incision was made with sufficient coverage to expose the injected filler to the forehead and temple. The skin was retracted laterally after three incisions were made. Next, the exposed frontalis muscle and SMAS were retracted inferiorly in the same range in the same manner. The frontalis muscle and SMAS were retracted into a single layer after dissection.

### 2.3 Three‐dimensional scanning to compare the change in appearance of the forehead and temple regions

As explained by Lee et al.,^16^ three‐dimensional (3D) scanning was carried out. Prior to injection, the initial 3D scanning operation was carried out to capture the volunteer's entire forehead and temple appearance. Structured light was used to scan it from the frontal and bilateral oblique angles. Software called Morpheus Plastic Solution Version 3.0 was used to recreate the scanned face. Through geometric analysis at three sites (lateral canthus, alare, and cheilion) bilaterally, the scanned images from the frontal and bilateral oblique aspects of the face were combined and merged. Volunteers were scanned again immediately after filler was injected into the bilateral forehead and temple regions, and 12 weeks later. The non‐injected side of the face, such as the mouth or nose, was used as a landmark for superimposition. Finally, 3D scanned facial images of the face before, after and 12 weeks later injected filler were superimposed to detect the change with filler injections.

## 3 RESULTS

US images were taken when the cannula was injected, releasing the filler into the forehead and temple regions of the cadaver (Figure 2). When the injection proceeded from the entry point of the STL to the forehead, the cannula was placed above the periosteum. It was also shown that the injected filler was distributed between the periosteum and frontalis muscle. When injecting into the temple region at the same entry point, both the cannula and injected filler were positioned exactly above the DTF. This image shows that the cannula wasn't inserted deeper than the DTF using the new injection method. As a result, the filler is consistently injected into the space between the DTF and subSMAS fat.

**FIGURE 2:**
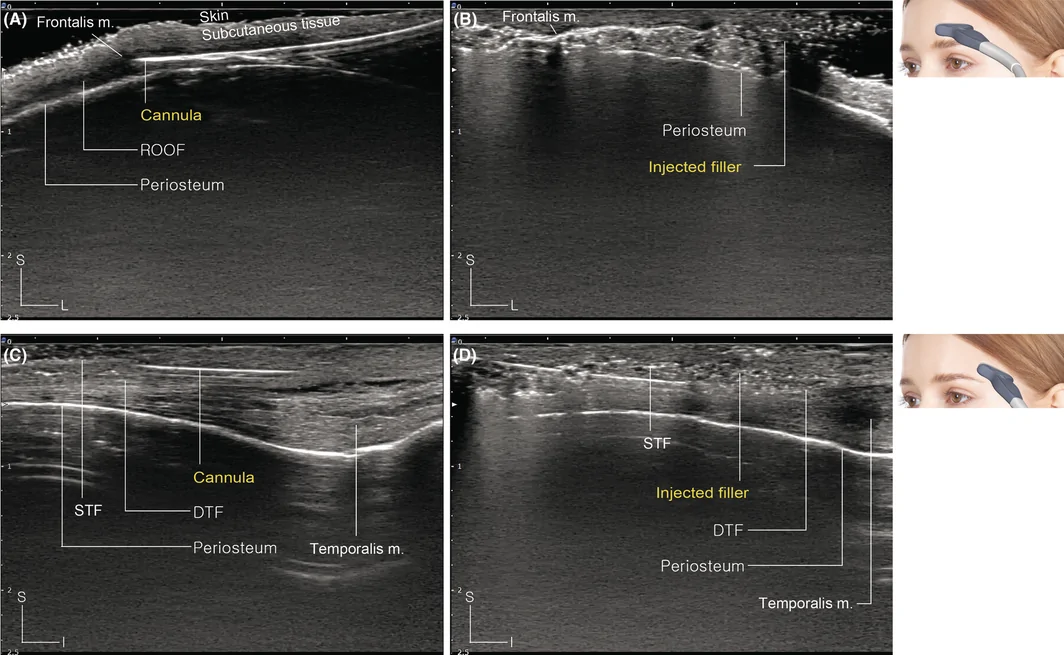
Ultrasonographic images of the cadaveric filler injection. (A) The cannula detected by ultrasonography is below the ROOF layer of the forehead. (B) The injected filler is detected on the supraperiosteal layer of the forehead. (C) The cannula is on the supra‐DTF layer of the temple. (D) The injected filler is on the supra‐DTF layer of the temple. ROOF, retro‐orbicularis oculi fat; DTF, deep temporal fascia; S, superficial; L, lateral; I, Inferior.

Such a filler distribution can be seen more clearly by dissecting the injection region (Figure 3). First, when the injection region was observed after the skin from the forehead to the temple was dissected, the dye‐mixed filler was covered with SMAS layers and a very thin frontalis muscle. Next, when the frontalis muscle of the forehead and the SMAS of the temple were retracted, the dye‐mixed filler was revealed. The injected filler was distributed continuously from the forehead to the temple around the injection entry point of the STL.

**FIGURE 3:**
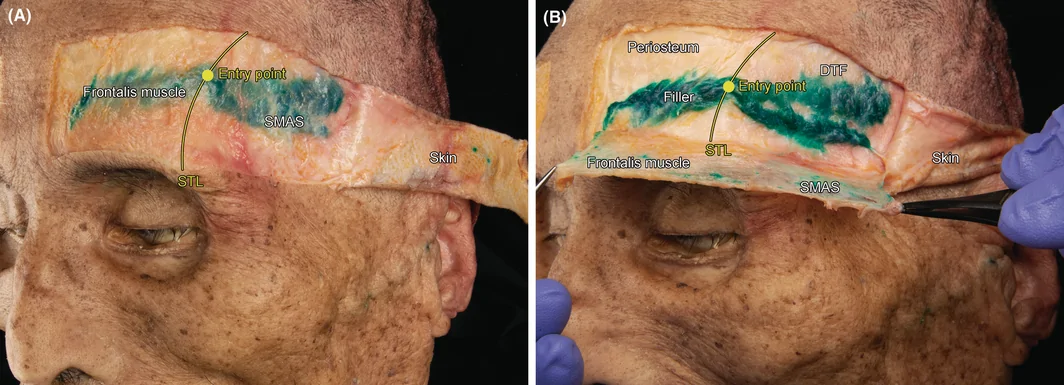
Two steps of cadaveric dissection for detecting the dye‐mixed filler. (A) The dissected skin is retracted. The dye‐mixed filler is not clearly visible because it is covered by the frontalis muscle and SMAS; however, it is distributed from the forehead to the temple. (B) The frontalis muscle and SMAS are retracted together. The clear shape of the dye‐mixed filler distributed in the supraperiosteal layer of the forehead and the supra‐DTF layer of the temple is revealed. The yellow line shows STL, and the yellow point shows injection entry point. SMAS, superficial musculoaponeurotic system; ROOF, retro‐orbicularis oculi fat; STL, superior temporal fusion line; DTF, deep temporal fascia.

Figure 4 shows images of a volunteer's face scanned from bilateral oblique aspects before, immediately after, and 12 weeks after the injection. The 3D models scanned each time were subsequently superimposed. Compared with the image before injection, the volume of the forehead and temple increased from a minimum of 2 cm to a maximum of 3.5 cm immediately and 12 weeks after injection. Particularly, after 12 weeks, the injected filler was evenly distributed, and the volume range increased owing to broadening of the filler. Based on these changes, the volume of the forehead significantly increased compared with that immediately after the injection.

**FIGURE 4:**
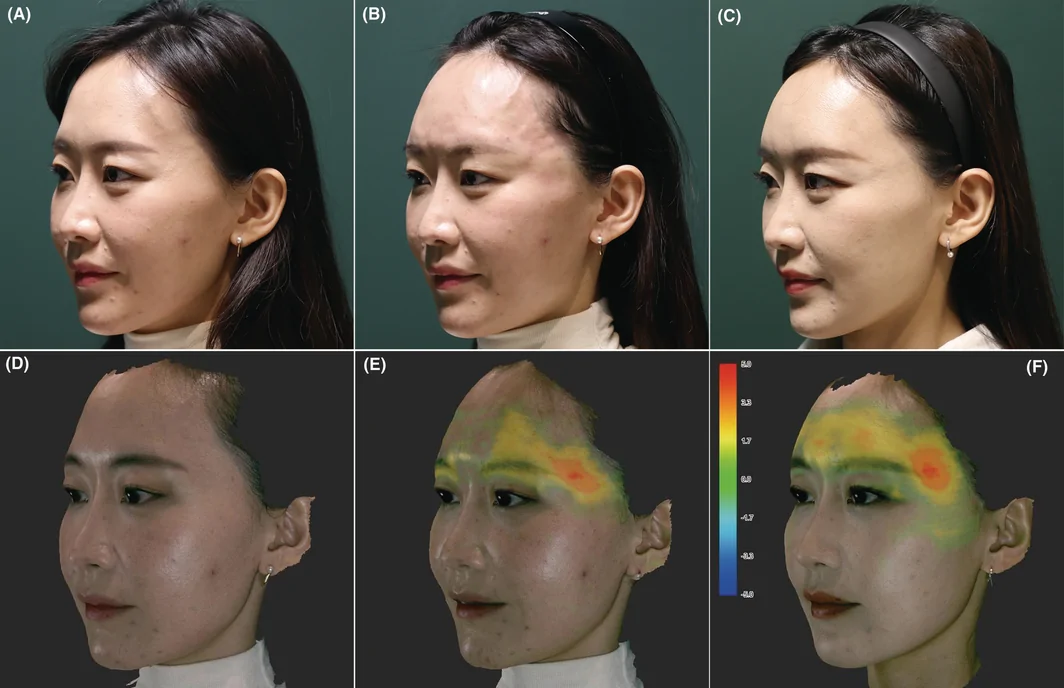
Comparison of augmentation according to filler injection using the photographs and three‐dimensional (3D) scan models of a 33‐year‐old female volunteer. Photographs of volunteer before (A), immediately after (B), and 12 weeks after the filler injection procedure (C). The following are images of the 3D model scanned before the filler injection (D), the 3D model scanned immediately after the injection (E), and the 3D model scanned 12 weeks after the injection (F). The 3D models immediately after injection (E) and 12 weeks after injection (F) are superimposed with the 3D model before injection (D), and the changed region is shown as a colormap. The range of the designated colormap is shown from −5 mm (blue) to +5 mm (red) based on the 3D model before injection.

Panoramic US images were obtained from the forehead to the temple in the volunteer (Figure 5). The US images also showed similar changes to the superimposed images. The images showed that the hypoechoic filler was distributed in the same layer from the forehead on the medial side of the STL to the temple on the lateral side. This filler was distributed in the superficial layer compared with the periosteum from the forehead and the DTF from the temple. Furthermore, although the cannula was injected in both directions from one entry point of the STL, the filler was connected to the area with the STL without breaking, and a single soft volume was formed. This shape of the spread filler was the same immediately after injection and after 12 weeks. Moreover, after 12 weeks of injection, the filler was spread evenly, and a smoother shape was formed. These results indicate that the proposed injection method is highly effective.

**FIGURE 5:**
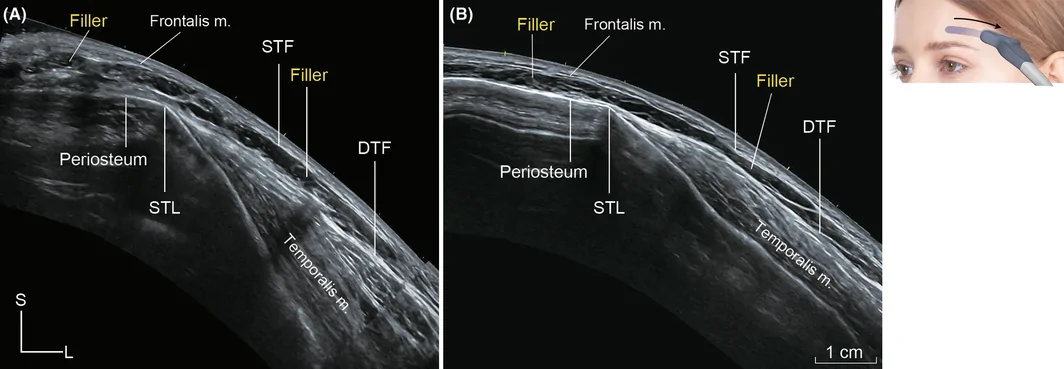
Panoramic ultrasonographic images of volunteer injected filler. (A) This is an image of a panoramic ultrasonographic image taken from the forehead to the temple immediately after injecting the filler. (B) This is an ultrasonographic image of the same region 12 weeks after filler injection in the same way. The filler is distributed continuously from the forehead to the temple in both immediately after and after 12 weeks, and the filler is forming more evenly after 12 weeks. STL, superior temporal line; DTF, deep temporal fascia; S, superficial; L, lateral.

## 4 DISCUSSION

This study presents a safe and easy cannula‐injection method for both the forehead and temple regions using a single‐entry point. For augmentation of depressed temples, filler injection is often performed only in the temple region. However, to form a natural and smooth facial curve, it is important to inject fillers into both the forehead and temporal region. Thus far, the structures of the forehead and temple have been considered to be completely separate, and for this reason, the injection entry points for each region have been set at separate locations. This method of injection can be burdensome for the patient, and satisfaction with the procedure can vary depending on the clinician performing the procedure. In addition, in rare cases, there is the potential for problems such as blindness and embolization due to damage to blood vessels in the forehead and temples during the procedure.^17^, ^18^, ^19^, ^20^, ^21^ However, according to the method presented in this study, the burden of the patient is reduced because only one entry point is used, and the procedure can be performed more easily regardless of the experience or skill level of the clinician.

In this study, the results of filler injection in the forehead and temple using a new injection method were anatomically shown through a cadaver. When the forehead and temple regions of the cadaver were observed using ultrasonography after injecting the filler, the filler was distributed in the supraperiosteal and supra‐DTF layers, which are loose connective tissues in each region. Additionally, the injected area was dissected layer‐by‐layer to check if the injected filler was precisely distributed below the ROOF and subSMAS fat. Through cadaver examination, it was confirmed that precise injection was possible in the chosen layer (supraperiosteal and supra‐DTF layers) of the forehead and temple region where the new injection method was used. Furthermore, the results on the volunteer clearly show the effectiveness of the injection. The effectiveness of injection was assessed by 3D modeling of the forehead and temple region before, immediately after, and 12 weeks after the procedure, and ultrasound imaging of the entire cross‐section. This procedure immediately increased the volunteer's forehead and temple volume, as had already been shown on cadaveric examination. What is particularly notable from a clinical viewpoint is that the filler, which was injected in a relatively rough shape, was improved and maintained in a smoother shape over time, as shown in the results after 12 weeks. These results indicate that the proposed injection method is highly effective. And both clinicians and patients expressed satisfaction when using the new injection method.

Figure 6 is an illustration showing how the filler injected by the proposed method in this study is anatomically located in the forehead and temple. The appropriate injection entry point suggested by the study is referenced on the STL. On this line, 1 cm above the frontotemporale can be designated as an optimal entry point. This point was determined by fully understanding the relationship between the forehead and temple anatomically and considering what would be most efficient for the patient and the clinician. In this study, ROOF and subSMAS fat are considered particularly key. Because the two structures are loose connective tissues in each region, they can be good target layers for filler injection. Since the supraperiosteal layer is in the layer just between the ROOF and periosteum, it is an especially easy structure for cannula injection. In contrast, subSMAS fat is in the middle of the complex layers of the temple. However, a relatively hard DTF, which was observed as the same layer with the periosteum of the forehead, is located in the layer just below the subSMAS fat, there is little risk of puncture unless a strong force is applied when injecting a non‐pointed needle, such as a cannula. By using such features of the forehead and temple, it is possible to inject it into the temple region much more easily than before. Based on these anatomical relationships, injection form the new entry point to the temple region could be performed in the same manner as for the supraperiosteal layer of the forehead. After insertion of the cannula until the periosteum was touched, the cannula could easily reach the subSMAS fat of the temple when inserted in the direction of the temple along the loose connective tissue layer. Finally, the injected filler is then neatly connected in both regions and placed beneath the frontalis muscle and superficial temporal fascia, as shown in Figure 6.

**FIGURE 6:**
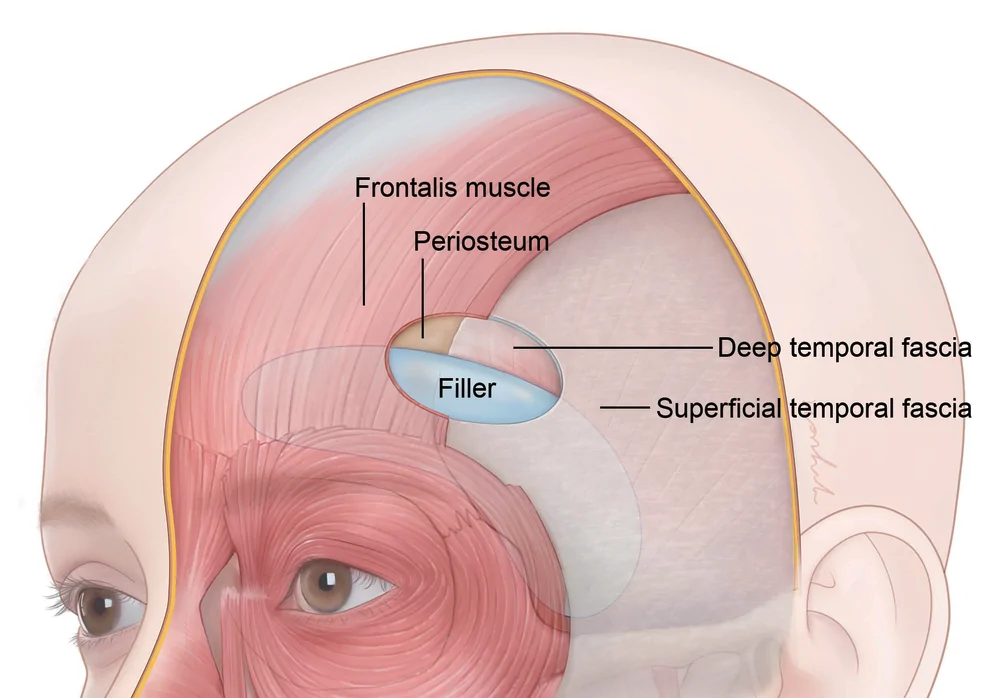
An illustration of the filler layer and range injected according to the new filler procedure. The injected filler forms a volume at a deeper layer than the frontalis muscle and SMAS. In the forehead, it is located between the frontalis muscle and the periosteum, and in the temple, it is located between the SMAS and DTF; that is, the filler was injected on the supraperiosteal layer and supra‐DTF layer below the ROOF and subSMAS fat. SMAS, superficial musculoaponeurotic system; DTF, deep temporal fascia; ROOF, retro‐orbicularis oculi fat.

## 5 CONCLUSIONS

In this study, the presented method emphasizes that there is only one entry point. This method can reduce patient pain and minimize externally visible wounds caused by injections. In addition, because the depth of injection can be determined based on the contact of the cannula with the bone during the procedure, it is possible to safely inject at a constant depth without requiring specific skills. This method is expected to become universal owing to the minimization of the burden on both the patient and clinician.

## AUTHOR CONTRIBUTIONS

All authors have reviewed and approved the article for submission. Conceptualization, Hyo‐Sang Ahn, Hyoung Moon Kim, Wook Oh. Writing—Original Draft Preparation, Hyo‐Sang Ahn, Kyu‐Ho Yi, Jisoo Kim, Hee‐Jin Kim. Writing—Review & Editing, Hyo‐Sang Ahn, Kyu‐Ho Yi, Jisoo Kim, Hee‐Jin Kim. Visualization, Hyo‐Sang Ahn, Kyu‐Ho Yi, Jisoo Kim, Hee‐Jin Kim. Supervision, Hee‐Jin Kim.

## FUNDING INFORMATION

This study was supported by Joonghun Pharmaceutical co., ltd (2021‐31‐1080).

## CONFLICT OF INTEREST STATEMENT

The authors declare no conflict of interest. The funding sponsors had no role in the design of the study, in the collection, analyses, or interpretation of data; in the writing of the manuscript, and in the decision to publish the results.

## ETHICS STATEMENT

This study was approved by the Institutional Review Board (IRB) of Yonsei University (IRB no. 2–2021‐0078), and all examinations were performed in accordance with the principles of the Declaration of Helsinki.

## Data Availability

The data that support the findings of this study are available from the corresponding author upon reasonable request.
